# Influence of Hot and Cold Environments on the Regulation of Energy Balance Following a Single Exercise Session: A Mini-Review

**DOI:** 10.3390/nu9060592

**Published:** 2017-06-10

**Authors:** Keyne Charlot, Cécile Faure, Sophie Antoine-Jonville

**Affiliations:** 1 Département Environnements Opérationnels, Institut de Recherche Biomédicale des Armées, 1 place Général Valérie André, BP 73, 91223 Brétigny-sur-Orge, France; keynecharlot@gmail.com; 2 Laboratoire des Adaptations au Climat Tropical, Exercice et Santé, EA3596, Université des Antilles, Pointe-à-Pitre, BP 250, 97157 Pointe-à-Pitre CEDEX, Guadeloupe, France; cecilefaure2203@gmail.com

**Keywords:** physical exercise, appetite, energy intake, eating behavior, extreme environment, gut hormones

## Abstract

Understanding the regulation of human food intake in response to an acute exercise session is of importance for interventions with athletes and soldiers, as well as overweight individuals. However, the influence of hot and cold environments on this crucial function for the regulation of body mass and motor performance has not been summarized. The purpose of this review was to exhaustively search the literature on the effect of ambient temperature during an exercise session on the subsequent subjective feeling of appetite, energy intake (EI) and its regulation. In the absence of stress due to environmental temperature, exercise-induced energy expenditure is not compensated by EI during an ad libitum meal following the session, probably due to decreased acylated ghrelin and increased peptide tyrosine tyrosine (PYY), glucagon-like peptide 1 (GLP-1), and pancreatic polypeptide (PP) levels. No systematic analysis has been yet made for major alterations of relative EI in cold and hot environments. However, observed eating behaviors are altered (proportion of solid/liquid food, carbohydrate/fat) and physiological regulation appears also to be altered. Anorexigenic signals, particularly PYY, appear to further increase in hot environments than in those that are thermoneutral. Ghrelin and leptin may be involved in the observed increase in EI after exercise in the cold, in parallel with increased energy expenditure. The potential influence of ambient thermal environment on eating behaviors after an exercise session should not be neglected.

## 1. Introduction

Understanding how the energy balance is altered by exercise may improve the care of two populations: those who want to lose fat and body mass, thus seeking a negative energy balance (overweight/obese population), and those who need to maintain an equilibrium between energy intake and expenditure (moderate to high-level athletes and soldiers). 

Concerning the first context, the rising worldwide incidence of obesity has become a major public health issue [1,2]. The simplest way to explain this epidemic is our modern way of life, resulting in a positive energy balance. Indeed, on one side, energy intake (EI) has increased owing to the large availability of highly-caloric foods. On the other side, spontaneous energy expenditure (EE) has decreased for several reasons including the development of motorized transportation and professional tasks that mostly require a seated position [3]. The effect of healthy diets on preventing weight (re)gain or reducing body mass in overweight/obese individuals has been extensively studied and their benefits and main limits (weight regain after drastic diets undertaken to lose body mass) are well-known [4]. Another way to induce a negative energy balance is to increase EE, possibly through voluntary physical activity (PA) [5]. However, it is necessary that increased in EE due to PA is not compensated by an increase in post-exercise EI. In contrast, athletes and soldiers need to carefully monitor EI to avoid a severe negative energy balance that compromises the quality of their training/preparation and therefore their performance [6,7]. Under extreme conditions, total EE substantially increases due to a large increase of PAEE (the physical activity-induced energy expenditure). Thus, total EE may be up to three times greater in high-level athletes during a period of competition than during recovery periods [8]. It is almost doubled in soldiers during operational deployments [6]. 

Athletes and soldiers expect increased PAEE to be compensated to avoid caloric deficit, but overweight and obese individuals count on the absence of PAEE through the decrease or absence of modifications of post-exercise EI to preserve a hypothetical chronic negative energy balance. It is therefore important to know whether PAEE is compensated or not by unconscious increases in post-exercise EI. The effect of a single exercise session on the components of energy balance regulation (subjective appetite, EI, plasma hormone levels related to eating behavior) has been well-documented and reviewed [9,10,11]. In these protocols, EI was assessed using either an ad libitum buffet (access to a variety of foods with varying amounts of protein, lipid, and carbohydrate) or a fixed meal (with one to three imposed dishes/foods). The reproducibility of EI derived from these meals was considered to be good [12,13,14]. The total EI of the control session (without exercise) was then compared to the interventional session (with exercise before the meal). Moreover, subjective appetite was assessed using visual analogue scales. Subjects had to answer the question ‘Do you feel hungry?’ by placing a vertical dash on a 100-mm horizontal scale, the left and the right extremities indicating ‘not at all’ and ‘extremely’, respectively. The distance from the extreme left of the scale to the subject’s vertical dash represented the rating score, expressed in mm. Satiety, fullness, or prospective food consumption could also be assessed with these scales. These scores were considered to be reliable for appetite research [15]. Although it seems that inter-individual responses to a single exercise session varied widely [16,17,18], a meta-analysis showed that PAEE is, at best, slightly compensated [10]. Thus, the relative energy intake (REI = EI − PAEE) was generally lower after an exercise session than after rest. This negative energy balance is conserved on a weekly [19] and monthly basis [20,21]. Indeed, there are models to estimate the percentage of fat mass loss based on weekly EE [22]. It is therefore common to observe EE exceeding EI in athletes [23] and soldiers [6]. The absence of energy compensation through changes in EI might be partially explained by acute changes in hormonal regulation of EI, decreased orexigenic hormone levels (total and/or acylated ghrelin; the acylated form being the post-translationally modified form of this gut peptide essential for its appetite-stimulatory effects), and/or increased anorexigenic hormone levels (leptin, peptide tyrosine tyrosine (PYY), pancreatic polypeptide (PP), cholecystokinine (CCK), and glucagon-like peptide 1 (GLP-1)) [11].

Most studies have been carried out in a neutral temperature (20–25 °C). Thermoregulatory processes are not extensively solicited in a temperate environment and are therefore unlikely to interfere with the proper regulation of other functions. Seasonal weather changes (for the general population as well as for athletes/soldiers) or the location of competitions/missions (for athletes/soldiers) can often subject these populations to adverse environments. Maintenance of the core temperature requires additional energy for thermoregulation during and after exercise if environmental temperatures are outside the thermoneutral zone, either below (cold) or above (warmth/heat) [24]. Indeed, the effects of long-term exposure to extreme environments on energy balance components are well-studied in mammals [25,26,27,28,29]: EI increases to compensate for higher EE to maintain internal temperature in cold environments and EI and EE both decrease in hot environments to avoid heat production and any subsequent increase in internal temperature. Moreover, thermoregulation and the regulation of food intake are both controlled by the hypothalamus [30,31,32], suggesting a strong interaction between these two systems. These environmental conditions should theoretically alter the well-known effects of PA on energy balance. Knowing the effects of these two forms of thermal strain during exercise, which is itself a stress, that disturb energy balance by increasing EE would allow a better understanding of how thermal environments influence EI after an exercise session. This mini-review presents the current knowledge in this field.

## 2. Effect of Exercise Sessions under Hot Conditions

Heat (or warmth) is used to designate higher than neutral temperatures. English nomenclature states that heat is more extreme than warmth. However, the objective threshold (in temperature and hygrometry) between a hot and warm environment is not established. Thus, environmental conditions might be described as warm in one study, whereas the same conditions might be described as hot in another. For simplicity, all conditions higher than neutral temperature (20–25 °C) were considered to be hot in this mini-review. Moreover, hygrometry is a major determinant of heat stress. Wet-bulb globe temperature (WBGT) (derived from temperature, humidity, and solar radiation) is widely used in workplaces, the military, and athletic organizations to determine conduct guidelines. Hygrometry is a major determinant of WBGT and small differences would induce non-negligible changes in WBGT and thus heat stress. The influence of hygrometry on thermoregulation processes during exercise is well-known, but its sole influence on modifying eating behavior after exercise has never been assessed. Thus, this aspect will not be addressed in this review. Table 1 presents the modalities of the studies that used heat as an environmental condition. It includes information on the participants, environmental conditions, exercise, test-meals, and the measurements performed. 

### 2.1. Effect on Subjective Feeling of Appetite and Energy Intake 

Shorten et al. [33] were the first in 2009 to assess the effects of heat in humans during exercise on appetite and EI. Only the thermoneutral exercise session increased EI (5193 ± 1597 kJ) relative to the control (rest) session in thermoneutral conditions (25 °C, 3743 ± 1150 kJ. Although EI after exercise in hot conditions (4326 ± 1677 kJ) was not significantly different from the control or exercise session in thermoneutral conditions, the REI was only lower in the hot session, suggesting that exercising in a hot environment induced a negative energy balance more efficiently than exercising in a temperate one. Macronutrient intake (carbohydrate, fat, and protein) was similar following the hot session for the two others. Solid food and carbohydrate intake were higher following the thermoneutral exercise session than the resting control session realized in the same environment. 

Three years later, Wasse et al. reported that EI after exercise in hot environment was lower (−1400 ± 2401 kJ), although not significantly (*p =* 0.08), than the same exercise in a thermoneutral environment [34]. This difference was similar for both the test meals initiated 2 and 5.5 h after exercise, suggesting that the effect of exercising in hot conditions might last up to two meals after the session. It is possible that the difference observed for the second meal was attributable to heat exposure itself (independently of exercise), since the meal was consumed a long time after the exercise and the subjects were exposed to heat throughout the recovery. Indeed, the acute effects of exercising in the heat might have vanished. However, several groups have observed post-exercise modifications in EI up to 24 h after the exercise session [16,35,36]. Moreover, heat exposure alone was insufficient to alter EI [37]. Macronutrient repartition was unfortunately not measured in this study. It might have been informative to know whether exercising in hot conditions can modify food preferences in a context where EI was significantly influenced by heat. In the same study, subjective ratings of hunger and prospective food consumption (using visual analogue scales) during the preprandial phase (two hours between the end of exercise and starting the first meal) were lower in the hot session than in the thermoneutral one, suggesting that heat induced a transient anorexigenic effect. This is consistent with the EI results. More recently, Kojima et al. [38] showed that exercise-induced anorexia persisted 15 min after the cessation of exercise in hot conditions, whereas this effect was not significant after exercise in thermoneutral conditions [38]. However, hunger or “motivation to eat” ratings were similar before, during, and after exercise in both sessions. They chose to not assess post-exercise EI during a test meal. It is impossible to know whether this anorexia was simply transient or would have lasted until the next meal, affecting EI. Finally, Faure et al. designed a protocol in which, for the first time, the sole effect of heat exposure and the combined effect of heat and physical exercise were assessed [37]. The REI after both exercise sessions (at 22 and 31 °C) was less than after the rest sessions (at 22 and 31 °C), indicating an absence of energy compensation. However, the absolute EI was strictly identical between sessions. Moreover, appetite scores were not altered by temperature or physical activity during the interventions, recovery phase, or at the beginning of meals. 

Exercising in hot conditions does not seem to alter or reduce EI relative to exercising in thermoneutral conditions. In some studies, a larger negative energy balance was observed following exercise in hot conditions than after thermoneutral conditions and subjective feelings of appetite also seemed to reflect this potential anorexigenic effect. Additional studies are needed to confirm whether heat increases the exercise-induced negative energy balance. The differences in study design may partially explain the absence of consensus. 

### 2.2. Effect on Appetite-Regulating Hormones 

The role of peripheral hormones on the regulation of food intake has been extensively described elsewhere [39]. Simply out, anorexigenic hormones secreted by small intestine (PYY, PP, CCK, and GLP-1) and adipose tissue (leptin), and orexigenic hormones secreted by the stomach (total and/or acylated ghrelin) and adipose tissue (adiponectin), send information to the hypothalamus to inhibit or stimulate food consumption. Leptin is also secreted by the stomach [40] and gastric leptin seems to act more rapidly than leptin secreted by adipose tissue. Three of the four studies presented in the previous section measured some of these hormonal responses to exercise in hot conditions. Plasma levels of total and/or acylated (its active form) ghrelin were not different after exercise in a hot environment that after the same exercises performed in a thermoneutral one [33,34,35,36,37]. Tomasik et al. [41] reported that heat exposure (30 °C) only increased plasma ghrelin levels compared to the thermoneutral condition [41]. The study of Faure et al. [35] consisted of two sedentary sessions in hot or neutral conditions (31 and 22 °C, respectively) that were comparable to the conditions of the study of Tomasik et al. [41]. However, total ghrelin levels were unaffected. This absence of a specific environment-related effect was also found with adiponectin [1], as well as with the anorexigenic hormones (leptin [1,33], PP [33,37], and CCK [37]). Only PYY appeared to be sensitive to exercise in hot conditions. Shorten et al. [33] observed higher plasma concentrations of PYY after exercising in the heat than during the rest session just before starting the post-exercise meal, whereas exercising in neutral conditions did not induce these changes. Moreover, PYY concentrations were higher during the postprandial period than those during both the rest and the exercise sessions in thermoneutral conditions. Kojima et al. [38] did not find a difference in PYY levels between exercise sessions in different thermal conditions for slightly shorter and less intense exercise sessions. However, no rest session was used in this study and no postprandial measurements were taken. 

Evidence of the effects of heat on exercise-induced modifications of appetite-regulating hormones is sparse. However, this effect appears to be minor, but leans toward an anorexigenic effect (via a possible increase in PYY levels) that is compatible with the likely decrease in EI after exercising in the heat. 

### 2.3. Specific Effect of Dehydration 

Control of the hydration status is fundamental when studying exercise in a hot environment. Indeed, dehydration is likely to be more pronounced after exercise in hot conditions than in thermoneutral conditions if water is not provided. If a meal is served after these sessions, it is possible that the differences in hydration levels and the sensation of thirst would interfere with the sensation of hunger, food choices (drinks instead of solid foods), and total EI. Three strategies are generally followed for studies to assess eating behavior: (1) compensate exercise-induced water losses so that the theoretical hydration status is the same in all conditions, (2) withhold water after exercise, the hydration status being different between conditions during the test meal, or (3) allow the participants to drink water ad libitum during and/or until the meal. In the first case, the sole effect of heat and/or exercise is theoretically assessed. In the second case, the additional effect of dehydration-induced thirst might confound the aforementioned effects, a point that was addressed in a letter [43]. In the third case, participants are likely to have a different hydration status at the beginning of the meal, even if unconscious water compensation occurred. Water loss after exercise was not compensated in three of the four studies presented in this review [33,34,37,38]. In two studies, water intake was forbidden either after exercise sessions [38] or until the meal [33]. In the last study, participants drank ad libitum [34]. It is likely that the effects of dehydration were mixed with those of temperature during exercise. Wasse et al. [34] reported that preprandial water intake was higher after exercising in the heat than in a thermoneutral environment, suggesting that subjects compensated the likely higher exercise-induced water losses in the heat, but no measurements were taken. Shorten et al. [33] found that water intake was higher after exercise sessions than after rest with no difference between exercise sessions performed in the heat and under thermoneutral conditions [33]. Surprisingly, the loss of body mass was identical (−0.65 kg) for the two exercise sessions, despite an 11 °C difference [33]. These sweat rates (0.98 L·h^−1^) are consistent with those generally observed [44], but a hot environment would be expected to have increased these rates [44]. The absence of a difference might be explained by the fact that the exercise volume and difference in temperature between the hot and thermoneutral sessions were too low to elicit significant differences in sweat loss rates. In the only study in which sweat loss was compensated before the test meal [37], water intake during the meal was very similar. This suggests that exercise and/or heat have no influence on subsequent water intake during the test meal if hydration is strictly controlled. 

Some studies have assessed the sole impact of dehydration on EI. Corney et al. manipulated 24-h food and fluid intake [45] or used exercise performed at 35 °C on the eve of the test meal [46], to induced hypohydration of −1.8% and 2.8%, respectively. Neither appetite ratings nor EI were different, even if subjective thirst and fluid intake were higher during the hypohydration sessions than during the euhydration sessions. Moreover, EI assessed during a test meal after exercise performed in a hypo or euhydrated state was also similar despite significant body mass losses after exercise (−0.28 for euhydrating and −2.32% for hypohydrating sessions) [47]. In this study, plasma levels of leptin, PYY, and PP were not sensitive to the hydration status. Only plasma ghrelin levels were lower in the hypohydrated state than in the euhydrated state. Thus, hydration status does not appear to effect appetite or EI. First, moderate exercise sessions lasting less than 60 min in the heat are unlikely to induce sweat rates that are different from those under thermoneutral conditions. Second, exercise-induced body mass losses differences of 1 to 2% are not sufficient to alter EI. However, severe dehydration (reduction of at least 4% of total body mass) that can occur in hot conditions in athletes during competition [48,49] or soldiers during operational missions [50] may alter EI [51,52].

## 3. Effect of Exercise Sessions under Cool Conditions

Exercise in cool conditions is either performed on land (between 8 and 12 °C) or in water (between 20 and 22 °C) (see Table 2 for details of the studies using cold as an experimental condition). This difference in temperature range is justified by the differences in thermal exchange between the environment (water or air) and the body [53]. Indeed, specific heat capacity and thermal conductivity and convection-induced thermal exchanges are much higher in water than in air due to higher density [54]. Thus, water provides the same cooling capacity as air that is 11 °C cooler [55]. Environmental temperatures corresponding to 20–25 °C on land and 30–35 °C in water are therefore considered to be thermoneutral. In the same way, water from 20 [56] to 22 °C [57] corresponds to air temperatures of approximatively 10 °C. We therefore consider exercise performed in warm water (33 to 34 °C) to not correspond to warm conditions, but rather to thermoneutral ones. 

### 3.1. Effect on Subjective Feeling of Appetite and Energy Intake 

In mammals, the cold season (winter for the northern part of the globe) is accompanied by an increase in body and fat mass mostly due to an increase in food intake [58]. In humans, these seasonal variations are also reported in various countries, despite plentiful access to food throughout the year [59,60]. This observation does not automatically concern indigenous populations living year-round in a polar climate that are characterized, although not systematically, by low fat mass [61,62]. Is this orexigenic effect also observable when applied during exercise? EI was found to largely increase after exercise in cool water [55,56]. In the study of White et al. [56], EI in this condition (3666 ± 1910 kJ) was 44% and 41% higher than that following the exercise session in thermoneutral water (2541 ± 849 kJ) and the rest session (2583 ± 1158 kJ), respectively. Dressendorfer [57] reported increases in EI of 171, 85% and 74% after exercise in cool water (2817 ± 201 kJ) relative to exercise in thermoneutral water (34 °C, 991 ± 96 kJ), on land (24 °C, 1455 ± 117 kJ), and the rest session (1542 ± 125 kJ), respectively. Exercising in cool water therefore induced energy compensation relative to exercising in thermoneutral water or on land.

Halse et al. [63] obtained results suggesting that immersion in water alone (cool or thermoneutral) stimulates EI [63]. After an exercise session (40 min at 70% VO_2max_), participants were either immersed in cool (15 °C) or thermoneutral water (33 °C) for 15 min before starting a buffet-type meal. EI increased following the two immersion sessions relative to the rest session without immersion (4089 ± 1585 kJ), regardless of the water temperature (4893 ± 1554 for cool and 5167 ± 1974 kJ and thermoneutral water). It is possible that immersion per se increased EI, although this increase may also be solely attributable to exercise. However, King et al. [64] found that 60 min of intermittent swimming in mid-cold/mid-thermoneutral water (~28 °C) did not alter EI assessed 7.5 h after exercise relative to the rest session [64]. Studies conducted on land avoid any effect of immersion on the sensation of appetite and EI. Kojima et al. [38] reported a stronger sensation of hunger during and after exercise in cool than neutral temperatures. This increase was, however, not significant in the study of Wasse et al. [34]. Nevertheless, changes in EI after exercising in the cold in the latter study, were consistent with those observed in immersion studies, EI still being higher after the session in the cold. Indeed, EI increased by 1450 ± 2345 kJ (*p =* 0.08) in young, lean, male subjects [34]. Interestingly, an 11% increase in EI was also observed in overweight males and females after a 45-min walk, corresponding to ~60% of VO_2max_, at 8 °C relative to those performing the same exercise at 20 °C, in the study of Crabtree & Blannin [65]. Thus, exercising in cool conditions may also stimulate EI and limit the exercise-induced negative energy balance in a population that may be seeking to lose weight. 

The methodology differed between the aforementioned studies. Moreover, the management of thermal comfort during and after exercise generally lacked a control. Clothing was either identical [56,65] or participants were left free to decide [34], depending on their comfort in the cold and the thermoneutral conditions. Others did not provide any details on clothing [57]. Greater homogeneity would have facilitated comparisons between studies. Nevertheless, EI always increased after exercising in cool conditions. This effect could be explained by modifications of plasma levels of eating behavior hormones. The robustness of this effect needs to be tested and the mechanism underlying it further investigated. 

### 3.2. Effect on Appetite-Regulating Hormones 

Hormonal measurements in these studies are sparse. Wasse et al. [34] and Kojima et al. [38] did not observe an effect of cold during exercise-induced modifications of ghrelin and/or PYY levels. However, Crabtree & Blannin [65] found that the area under the curve of acylated ghrelin (the active form of ghrelin) (from pre-exercise to the end of meal) was higher after exercising in the cool than under thermos conditions. This is concordant with the observed increase in EI, although the absence of a correlation between acylated ghrelin and the difference in EI between the two conditions weakens this link. Although total and acylated ghrelin concentrations were not affected by temperature (7, 20, and 33 °C) or exercise in a recent study [38], Tomasik et al. [41] reported higher total ghrelin concentrations after exposure to 2 °C relative to 20 °C. The subsequent EI was not assessed in these studies, but ghrelin could play a role in the increase in EI following exposure to the cold. 

Other evidence suggests an effect of cold on leptin levels. Zeyl et al. [66] showed that in vivo immersion in cold water (18 °C) or decreasing the temperature of incubated surgically removed human subcutaneous adipose tissue samples from 37 to 27 °C in vitro both decreased leptin levels. Leptin secreted by adipose tissue is known to control long-term regulation of EI [40]. However, gastric leptin produces a short-term effect to rapidly influence EI [40,67]. In theory, a decrease in leptin levels should stimulate EI [40] and could therefore partially explain the cold-induced increase in food intake. In their study, Laursen et al. did not observe a decrease in leptin concentrations in a cool environment [38], but the authors propose that their exposure period to the cold might have been insufficient. However, as already mentioned, it was found that a short cool or thermoneutral immersion after an exercise session in a thermoneutral environment increased EI [63]. 

In summary, the analysis of plasma hormone concentrations in the few available studies showed that acylated ghrelin levels increased during cold exposure whereas leptin levels decreased during immersion in cold water. It will be necessary to measure changes in leptin concentrations after exercising realized in cool and neutral environments to better assess the role that ghrelin and leptin may play in the regulation of short-term EI after exposure to the cold.

## 4. Conclusions, Limitations, and Perspectives 

**Figure 1:**
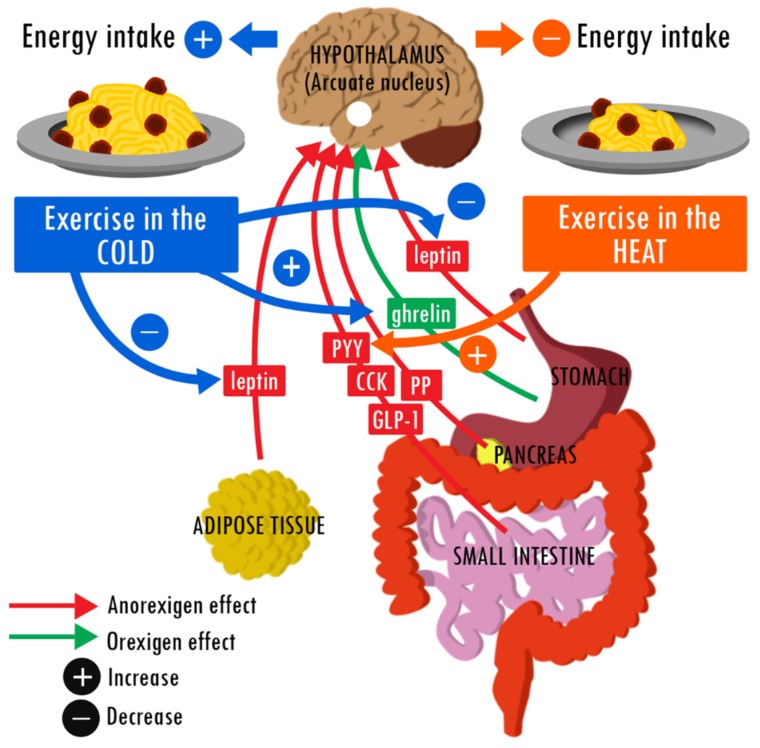
Hypothetical effects of heat and cold during a single exercise session on the regulation of food intake. Literature suggests that exercising under cool conditions induces an increase in post-exercise EI relative to the same exercise under thermoneutral conditions. This orexigenic effect could be explained in part by a decrease in plasma leptin and an increase in acylated ghrelin levels. Exercising in the heat may have an opposite effect, thus decreasing EI relative to exercising at a neutral temperature. The difference potentially involves an increase in plasma PYY levels.

Physical activity affects the phylogenetic development of most functions in mammals [68]. Thus, there is little chance of maintaining a stable body mass without sufficient PA [69]. Accordingly, regular PA limits the risk of becoming overweight [68] and is an efficient means to reduce body and fat mass in overweight/obese individuals [20,22]. In both cases, levels of markers of good health are also maintained or improved [70]. PAEE is only slightly compensated in thermoneutral conditions [10] and the organism uses energy from endogen stores (mainly adipose tissue) to ensure the total EE. Overall, the studies presented in this mini-review strongly suggest that this response is altered under less clement conditions. Figure 1 summarizes these hypothetical alterations. Heat can sometimes transiently decrease EI and enhance the PA-induced negative energy balance [33,34]. The only plausible explanation is a short-term heat-induced increase in the plasma levels of PYY, an intestinal anorexigenic hormone. On the other hand, the effect of cold appears to be more reproducible: all exercise sessions in cool water or under cool conditions on land led to an increase in post-exercise EI. To date, it is difficult to link this likely orexigenic effect to modifications of eating behavior hormone levels. Available evidence implies an increase in acylated ghrelin levels (the active form of the only orexigenic hormone) and a decrease in leptin levels (an anorexigenic hormone). 

These conclusions need to be put into context. First, the cautious tone used in this review ensues from the relatively small number of publications in this subject and the differences in observed responses characterized by high inter-individual variability [16,17,18]. The heterogeneity of the methodology of the studies (exercise modalities, environmental conditions, the nature of test meals, and samples characteristics) partially explains the difficulty in obtaining a consensus. Second, this mini-review focuses on acute effects and avoids mention of medium to long-term effects that would be significantly more enlightening in terms of the regulation of the energy balance. The reason is simple: this topic has never been studied. Despite these unavoidable limitations, this mini-review summarizes these very recent studies that present strong evidence and plausible hypotheses that require further examination, and target the hormones that are the most likely to be influenced by environmental conditions. It should help those working in the field of eating behavior to appreciate the importance of heat and cold in the regulation of the energy balance and to encourage them to consider testing thermoregulatory parameters, historically linked to research in exercise physiology, when conducting energy balance studies of appetite. 

There are two major perspectives for the future: (1) to first perform further studies, trying to reduce methodological heterogeneity, to strengthen the conclusions, and then (2) to consider the long-terms effects of exercising in cool or hot conditions on energy balance. This would allow an understanding of whether acute effects are maintained in the long term. A full understanding of the impact of environmental conditions will require observations for at least several days to several months to fully appreciate the impact on energy balance and define the potential actions to be taken, irrespective of the targeted population (athletes, soldiers, or overweight/obese individuals). Athletes and soldiers may face adverse environments during competitions/missions (due to seasonal weather changes or the locale). Athletes may even seek to acclimate to such environments by replicating them during training sessions [71]. Their shared aim is to maintain an EI that matches their EE to maintain their performances [6,7], an objective that may be harder to reach for soldiers in an operational context. It is likely that heat augments the exercise-induced decrease in relative EI whereas cold would limit it. It is premature to prescribe recommendations, but current knowledge may indicate that energy supplies should be carefully monitored under hot conditions to meet energy needs. Individuals aiming to prevent (re)gaining or reducing body mass use PA to induce a chronic negative energy balance. In this context, it appears that this objective will be more easily obtained under hot than cool conditions. Further studies are needed to demonstrate this conclusion. The use of thermal rooms to create a hot environment during training sessions would help ascertain whether heat can be a useful tool to stimulate weight loss.

There is a continuing need to better understand how hot and cold environments alter the regulation of energy balance following a single exercise session and during physical training. The articles presented in this mini-review suggest that thermal conditions influence appetite, EI, and hormones that modulate eating behavior after an exercise session.

## Figures and Tables

**Table 1:** Description of studies conducted in hot conditions.

Study	Participants	Temperature/Hygrometry	Exercise/Rest	Time between Exercise and Meals	Meals	Energy Intake (Absolute/Relative)	Subjective Feeling of Appetite	Hormones
Wasse et al. [34]	11 lean M	30 °C/50%	60 min 65% VO_2max_	120 and 330 min	Cold buffet	Yes/No	Yes	Acylated ghrelin
		20 °C/50%	60 min 65% VO_2max_					
Shorten et al. [33]	11 lean M	36 °C/30%	40 min 70% VO_2max_	45 min	Breakfast buffet	Yes/Yes	No	Acylated ghrelin, leptin, PP, PYY
		25 °C/30%	40 min 70% VO_2max_					
		25 °C/30%	Rest					
Kojima et al. [38]	11 lean M	36 °C/40%	30 min 65% VO_2max_		No meal		Yes	Total ghrelin, PYY
		24 °C/40%	30 min 65% VO_2max_					
Faure et al. [37]	10 lean M	31 °C/45%	40 min 60% VO_2max_	30 min	Sandwiches	Yes/Yes	Yes	Total ghrelin, PP, CCK
		31 °C/45%	Rest					
		22 °C/55%	40 min 60% VO_2max_					
		22 °C/55%	Rest					
Laursen et al. [42]	11 M	33 °C/60%	60 min 60% W_max_		No meal		No	Adiponectin, total and acylated ghrelin, leptin
		20 °C/60%	60 min 60% W_max_					

M = Males.

**Table 2:** Description of studies conducted under cool conditions.

Study	Participants	Temperature/Hygrometry	Exercise/Rest	Time between Exercise and Meals	Meals	Energy Intake (Absolute and Relative)	Subjective Feeling of Appetite	Hormones
Wasse et al. [34]	10 lean M	10 °C/50%	60 min 65% VO_2max_	120 and 330 min	Cold buffet	Yes/No	Yes	Acylated ghrelin
		20 °C/50%	60 min 65% VO_2max_					
White et al. [56]	11 lean M	33 °C (in water)	45 min 60% VO_2max_	45 min	Buffet	Yes/No	No	No
		20 °C (in water)	45 min 60% VO_2max_					
		25 °C/NC	Rest					
Dressendorfer [57]	6 lean M	34 °C (in water)	30 min 70% VO_2max_	NC	Sweet foods	Yes/No	No	No
		22 °C (in water)	30 min 70% VO_2max_					
		24 °C (on land)/NC	30 min 70% VO_2max_					
		24 °C/NC	Rest					
Crabtree et al. [65]	10 OW M & 6 OW F	8 °C/40%	45 min 55% VO_2max_	45 min	Buffet	Yes/Yes	No	Total and acylated ghrelin, PYY
		20 °C/40%	45 min 61% VO_2max_					
Kojima et al. [38]	11 M	12 °C/40%	30 min 65% VO_2max_		No meal		Yes	Total ghrelin, PYY
		12 °C/40%	30 min 65% VO_2max_					
Laursen et al. [42]	11 M	7 °C/60%	60 min 60% W_max_		No meal			Adiponectin, total and acylated ghrelin, leptin
		20 °C/60%	60 min 60% W_max_					

NC = Not communicated; M = Males; F = Females; OW = Overweight.
